# C-Terminal Helical Domains of Dengue Virus Type 4 E Protein Affect the Expression/Stability of prM Protein and Conformation of prM and E Proteins

**DOI:** 10.1371/journal.pone.0052600

**Published:** 2012-12-26

**Authors:** Wen-Yang Tsai, Szu-Chia Hsieh, Chih-Yun Lai, Hong-En Lin, Vivek R. Nerurkar, Wei-Kung Wang

**Affiliations:** 1 Department of Tropical Medicine, Medical Microbiology and Pharmacology, University of Hawaii at Manoa, Honolulu, Hawaii, United States of America; 2 Pacific Center for Emerging Infectious Diseases Research, John A. Burns School of Medicine, University of Hawaii at Manoa, Honolulu, Hawaii, United States of America; 3 Institute of Microbiology, College of Medicine, National Taiwan University, Taipei, Taiwan; University of Texas Medical Branch, United States of America

## Abstract

**Background:**

The envelope (E) protein of dengue virus (DENV) is the major immunogen for dengue vaccine development. At the C-terminus are two α-helices (EH1 and EH2) and two transmembrane domains (ET1 and ET2). After synthesis, E protein forms a heterodimer with the precursor membrane (prM) protein, which has been shown as a chaperone for E protein and could prevent premature fusion of E protein during maturation. Recent reports of enhancement of DENV infectivity by anti-prM monoclonal antibodies (mAbs) suggest the presence of prM protein in dengue vaccine is potentially harmful. A better understanding of prM-E interaction and its effect on recognition of E and prM proteins by different antibodies would provide important information for future design of safe and effective subunit dengue vaccines.

**Methodology/Principal Findings:**

In this study, we examined a series of C-terminal truncation constructs of DENV4 prME, E and prM. In the absence of E protein, prM protein expressed poorly. In the presence of E protein, the expression of prM protein increased in a dose-dependent manner. Radioimmunoprecipitation, sucrose gradient sedimentation and pulse-chase experiments revealed ET1 and EH2 were involved in prM-E interaction and EH2 in maintaining the stability of prM protein. Dot blot assay revealed E protein affected the recognition of prM protein by an anti-prM mAb; truncation of EH2 or EH1 affected the recognition of E protein by several anti-E mAbs, which was further verified by capture ELISA. The E protein ectodomain alone can be recognized well by all anti-E mAbs tested.

**Conclusions/Significance:**

A C-terminal domain (EH2) of DENV E protein can affect the expression and stability of its chaperone prM protein. These findings not only add to our understanding of the interaction between prM and E proteins, but also suggest the ectodomain of E protein alone could be a potential subunit immunogen without inducing anti-prM response.

## Introduction

Dengue virus (DENV) belongs to the genus *Flavivirus* of the family *Flaviviridae*. The four serotypes of DENV (DENV1, DENV2, DENV3, and DENV4) cause the most important arboviral diseases in the tropical and subtropical regions, including a debilitating disease, dengue fever, and a severe and potentially life-threatening disease, dengue hemorrhagic fever/dengue shock syndrome [1]–[3]. It was estimated that more than 2.5 billion people in over 100 countries are at risk of infection and more than 50 million dengue infections occur annually worldwide [1]–[3]. While considerable efforts have been made to develop therapeutic or prophylactic interventions, no antiviral or vaccine against DENV is currently available.

DENV contains a positive-sense, single-stranded RNA genome of approximately 10.6 kilobases in length. Flanked by the 5′ and 3′ untranslated regions, the genome contains a single open reading frame encoding a polyprotein, which is cleaved by cellular and viral protease into three structural proteins, capsid, precursor membrane (prM) and envelope (E), and seven nonstructural proteins [4]. DENV enters the cell through receptor mediated endocytosis [4]–[6]. After entry and uncoating of DENV, translation, genome replication and assembly occur in the membranes derived from endoplasmic reticulum (ER), where immature virions bud into the lumen of ER and transport through the secretory pathway [4], [5], [7], [8]. In the trans-Golgi, the prM protein is cleaved by furin or furin-like protease resulting in the formation of mature virions, though the cleavage is often inefficient [9]–[12].

**Figure 1:**
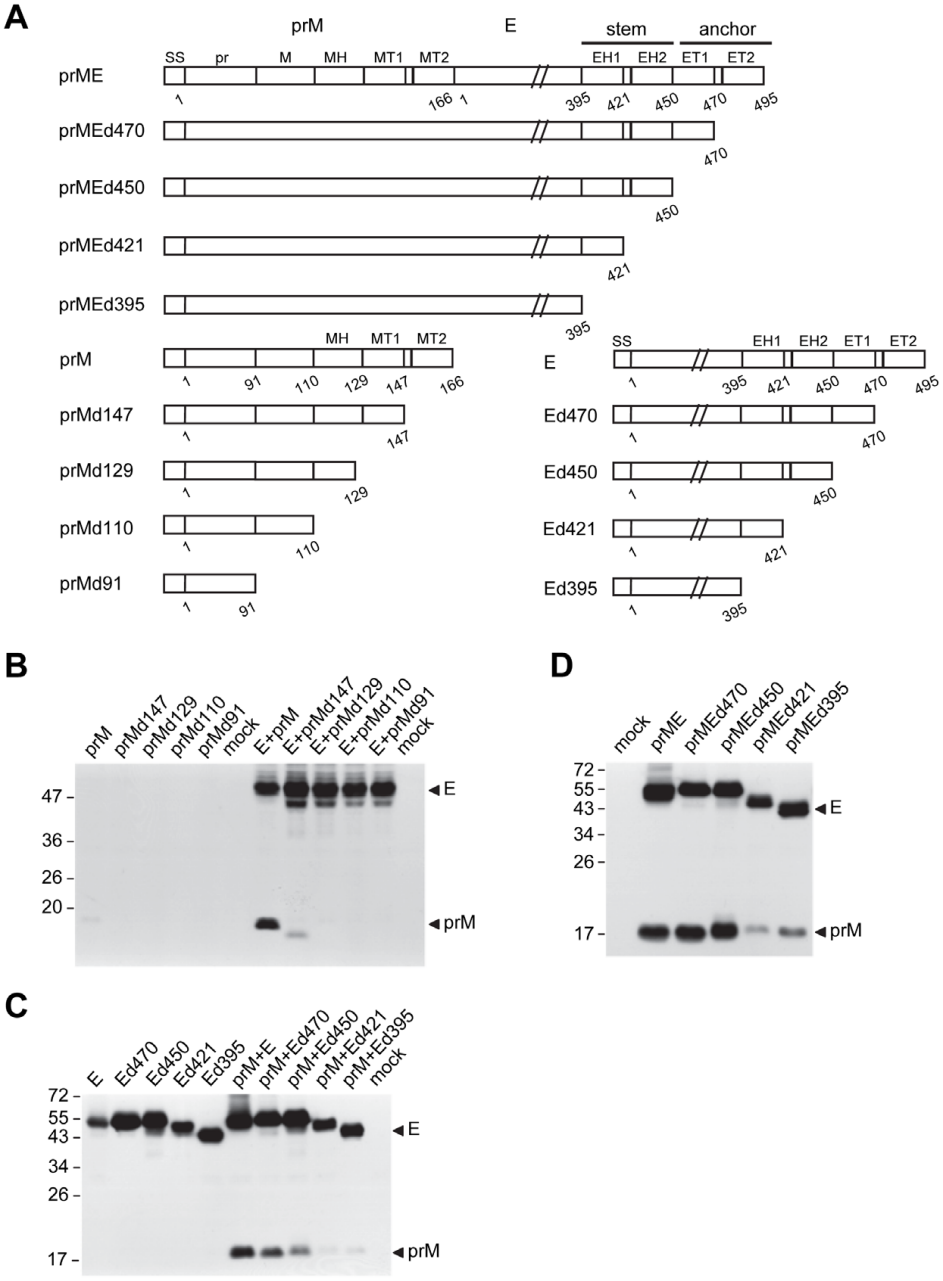
Schematic drawing of DENV4 prME, prM and E constructs with serially C-terminal truncation and their expression. (A) The C-terminus of E protein contains two α-helical domains (EH1 and EH2) in the stem region, followed by two transmembrane domains (ET1 and ET2) in the anchor region [18]. The C-terminus of prM protein contains an α-helical domain (MH) and two transmembrane domains (MT1 and MT2). WT and a series of C-terminal truncation of prME, prM and E constructs were shown. (B) 293T cells were transfected with prM and its C-terminal truncation constructs in the presence or absence of E construct. (C) 293T cells were transfected with E and its C-terminal truncation constructs in the presence or absence of prM construct. (D) 293T cells were transfected with prME and its C-terminal truncation constructs. Cell lysates collected at 48 h post-transfection were subjected to Western blot analysis using human serum of a confirmed dengue case [31], [32]. One representative experiment of three is shown. The size of molecular weight markers is shown in kDa. Arrow heads indicate E and prM proteins.

The E protein participates in virus entry and is the major target of neutralizing antibodies and vaccine development [4], [13], [14]. In the genus *Flavivirus*, there are several serocomplexes including DENV, Japanese encephalitis virus (JEV), and tick-borne encephalitis virus (TBEV) serocomplexes. Antibodies that recognize members from different serocomplexes, all/subset of members within a serocomplex and a single member are called flavivirus group-reactive (GR), complex/subcomplex-reactive (CR/sCR) and type-specific (TS), respectively [15]. The N-terminal ectodomain of E protein contains three domains (domains I, II and III) based on X-ray crystallographic studies [16]. The C-terminus of E protein contains two α -helices (EH1 and EH2) in the stem region and two transmembrane domains (ET1 and ET2) in the anchor region, which crosses the two leaflets of lipid bilayer [17], [18] (Figure 1A). Studies of TBEV revealed that both ET2 and ET1 were involved in the assembly of E protein into virus-like particles (VLPs) and the fusion step of virus entry, EH2 stabilized the prM-E heterodimer, and EH1 was involved in the irreversible trimerization of soluble E protein in low pH environment [17], [19]–[21].

The prM protein contains 166 amino acids; cleavage at position 91 results in the pr peptide and M protein. The C-terminus of prM protein contains a helical domain (MH) and two transmembrane domains (MT1 and MT2) (Figure 1A) [18]. After biosynthesis in the rough ER, prM and E proteins form a heterodimer, which was reported to be important for assembly of VLPs [17], [20]. During maturation, prM-E heterodimeric interaction in the immature virions and association of pr peptide with E protein (after cleavage of prM protein) in the mature virions could prevent premature fusion of E protein within acidic compartments along the secretory pathway [12], [21]–[23]. Studies of TBEV and JEV reported prM protein as a chaperone for proper folding of E protein probed by a single monoclonal antibody (mAb) [24], [25]. However, how prM protein affects the conformation of E protein recognized by different mAbs and whether E protein affects the expression, stability or conformation of prM protein remains largely unknown.

While a previous study of mice immunized with prM and M proteins reported a protective role of anti-prM antibodies against DENV infection [26], two recent studies revealed that anti-prM mAbs can enhance the infection of immature DENV particles by antibody-dependent enhancement (ADE) [27], [28]. Another study showed that human anti-prM mAbs did not neutralize DENV but potently promote the infectivity of immature DENV by ADE, suggesting anti-prM responses should be minimized in dengue vaccines [29]. Since the cleavage of prM protein during DENV maturation was inefficient [9]–[12], the presence of prM protein in dengue vaccine preparation (live attenuated or killed virus vaccine) could represent enhancing epitopes and be potentially harmful [29]. Considering the roles of prM protein as being a chaperone for E protein and preventing E protein from premature fusion, a better understanding of the prM-E interaction and the effect on the recognition of E and prM protein by different antibodies would provide important information for the design of subunit dengue vaccines to preserve neutralizing epitopes and remove non-neutralizing and potentially enhancing epitopes.

In this study, we investigated a series of C-terminal truncation constructs of DENV4 prME, E and prM, and found that E protein increases the expression of prM protein by maintaining its stability after synthesis; EH2 domain is critical. EH2 and ET1 domains are involved in prM-E interaction. PrM protein and truncation of EH2 or EH1 domain affect the recognition of E protein by several mouse and human mAbs. These findings have implications for the development of subunit vaccines against DENV.

## Methods

### Plasmid constructs

The prM/E expression constructs of DENV4 (pCB-D4, designated as prME in this study) was described previously [30]. A series of constructs were generated by PCR and cloning based on prME construct. The C-terminal truncation constructs of prME (prMEd470, prMEd450, prMEd421 and prME d395) contained the entire prM gene and truncated E gene at the corresponding amino acid position of 470, 450, 421 and 395, respectively (Figure 1A). The C-terminal truncation constructs of E and prM were shown in Figure 1A. All the constructs were confirmed by sequencing the entire inserts to rule out second site mutations. The details of PCR/cloning strategy and sequences of primers were summarized in Table S1. CD4D4SA, which contained the ectodomain of CD4 and the stem-anchor regions of DENV4 E protein, was described previously [31].

### Transfection, cell lysates, Western blot analysis and dot blot assay

293T cells (from ATCC) prepared in a 10 cm-culture dish at 5×10^5^ cells per dish one day earlier were transfected with 10 µg of plasmid DNA by calcium phosphate method [31]. At 48 h post-transfection, cells were washed with 1× PBS and treated with 1% NP40 lysis buffer (100 mM Tris [pH 7.5], 150 mM NaCl, 20 mM EDTA, 1% NP40, 0.5% Na deoxycholate and protease inhibitors [Roche Diagnostics]), followed by centrifugation at 20,000×g and 4°C for 30 min to obtain cell lysates [31]. For Western blot analysis, cell lysates were added to non-reducing buffer (2% SDS, 0.5 M Tris [pH 6.8], 20% glycerol, 0.001% bromophenol blue [final concentration]) and subjected to 12% polyacrylamide gel electrophoresis (PAGE), followed by transfer to nitrocellulose membrane, blocking and incubation with primary antibody (human sera from confirmed dengue cases) and secondary antibody [31], [32]. After final washing, the signals were detected by enhanced chemiluminescence reagents (Perkin Elmer life sciences). For dot blot assay, cell lysates in 1% NP40 lysis buffer were diluted in bromophenol blue containing 1×PBS and blotted by using a 96-dot formatted dot-blotter (Labrepco) to nitrocellulose membrane, followed by blocking, incubation with primary (mixed mouse mAbs or each mouse or human mAb) and secondary antibodies, and detection as described above [33]. For dots containing mixtures of native prM/E proteins in 1% NP40 lysis buffer and denatured prM/E proteins in reducing buffer (non-reducing buffer with 0.71 M β-mercaptoethanol [final concentration]), denatured prM/E proteins were first blotted, followed by washing with 1×PBS three times and blotting with native prM/E proteins. The intensities of the dots containing wild type (WT) E protein (expressed by prME) and mutant E proteins including E protein alone and different truncated E proteins in the presence or absence of prM protein were analyzed by ImageJ (NIH, Bethesda, MD) [33]. Two-fold serial dilutions of cell lysates derived from WT prME construct were dotted on each membrane and a linear decrease in intensity excluded the possibility of overexposure. The recognition index (R.I.) of a mAb to a mutant E protein = [intensity of mutant E dot/intensity of WT E dot] (recognized by a mAb) divided by [intensity of mutant E dot/intensity of WT E dot] (recognized by mixed mAbs) as described previously [33].

### Mouse and human mAbs

The mouse anti-E mAbs in this study included two GR mAbs (4G2 and DEN2-12), one CR mAb (DEN3-3), and two DENV4 TS mAbs (1H10-6-7 and 1H10-5-7) [33]. The human anti-E and anti-prM mAbs were derived from a case of primary DENV4 infection and a case of primary DENV3 infection, respectively, by memory B cell immortalization and cloning as described previously [34], [35]. The human anti-E mAbs included six GR mAbs (DVD19.4, DVD19.13, DVD23.3, DVD23.4, DVD26.3 and DVD26.11) and two DENV4 TS mAbs (DVD9,8 and DVD9.9). The human anti-prM mAbs included two CR mAbs (DVB59.3 and DVB18.5) and two sCR mAbs (DVB65.5 and DVB32.4) [35].

### Radioimmunoprecipitation and pulse-chase experiment

293T cells prepared in a 6-well plate were transfected with plasmid DNA by calcium phosphate method. At 20 h, cells were washed, incubated with methionine-free medium, followed by 50 µCi [^35^S] methionine (Amersham Biosciences) at 37°C for 6 h, and collected to obtain cell lysates [31]. Following pre-clear, cell lysates were incubated with mouse anti-E mAb FL0232 (Chance Biotechnology) or mixed human sera of conformed dengue cases at 4°C overnight and then with protein A sepharose beads (Amersham Biosciences) at 4°C for 6 h [31]. After washing, the beads were mixed with 2× sample buffer and heated, and the solubilized fraction was subjected to SDS-12% PAGE [31]. For the pulse-chase experiment, cells were incubated with methionine-free medium at 20 h post-transfection, pulsed with 60 µCi [^35^S] methionine at 37°C for 20 min, and chased at 0 min and 90 min; mixed human sera of confirmed dengue cases were used in the immunoprecipitation [33]. The intensities of the prM and E bands were analyzed by imageQant (GE Healthcare, UK); the relative prM/E expression (for pulse-chase experiment at 90 min) = [intensity of prM band/intensity of truncated E band] at 90 min divided by [intensity of prM band/intensity of truncated E band] at 0 min, and the prM/E index (for radioimmunoprecipitation) = [intensity of prM band/intensity of truncated E band] divided by [intensity of prM band/intensity of WT E band]. Notably, since there were 18 methionine residues in the WT E protein and 16, 15, 14 and 12 methionine residues in Ed470, Ed450, Ed421 and Ed395, respectively, the relative prM/E expression and prM/E indices for the truncated mutants were corrected by different factors accordingly. Two-tailed Mann-Whitney test was used to determine the difference in the relative prM/E expression at 90 min between WT and mutants by GraphPad Prism5 (GraphPad Inc., CA).

### Sucrose gradient sedimentation analysis

Plasmid DNA was transfected to 293T cells prepared in a 10-cm culture dish by calcium phosphate method. At 48 h post-transfection, cells were washed with 1× PBS, resuspended in 1× PBS, treated with 1% Triton X-100 on ice for 30 minutes, and then loaded into a 5 to 20% (wt/wt) sucrose gradient made with gradient buffer (50 mM Tris-HCl [pH 8.0], 150 mM NaCl, 2 mM EDTA, 0.5% Triton X-100, 1 mM phenylmethylsulfonyl fluoride) [31]. The gradient was ultracentrifuged at 247,606×g at 15°C for 22 h, and each of the 14 fractions was collected and subjected to Western blot analysis. Marker proteins (BioRad) were subjected to the same analysis.

### Enzyme digestion

Aliquots of total cell lysates at 48 h post-transfection, fraction 6 or fraction 14 from sucrose gradient sedimentation were treated with 500 U of endo-β-N-acetylglucosaminidase H (endo H) or peptide N-glycosidase F (PNGase F) at 37°C for 1 h according to the manufacture's instructions (New England Biolabs), and subjected to Western blot analysis.

### Capture ELISA

Flat-bottom 96 well plate was coated with a human dengue-immune serum at 4°C overnight, followed by blocking with 1% BSA in PBS for 1 h and addition of culture supernatants containing truncated E proteins (derived from prMEd421, prMEd395, Ed421 or Ed395) and known concentrations of recombinant DENV4 E protein, which was used to generate a standard curve. After addition of mixed mouse mAbs and anti-mouse IgG conjugated with HRP each at 37°C for 1 h, TMB substrate and stop solution, the absorbance at a wavelength of 450 nm (OD 450) with reference wavelength of 650 nm was read [33] and interpolated to determine the concentration of truncated E proteins (GraphPad Prism5, GraphPad software Inc., CA). Comparable amounts of truncated E protein (0.6 ng) were added to 96 well plate pre-coated with mixed mouse mAbs (for testing human mAbs) or human dengue-immune serum (for testing mouse mAbs), followed by addition of each human mAb or mouse mAb, secondary antibody, TMB substrate and stop solution, the OD 450 with reference wavelength of 650 nm was read [33].

## Results

### E protein increases the expression of prM protein and EH2 domain is critical

**Figure 2:**
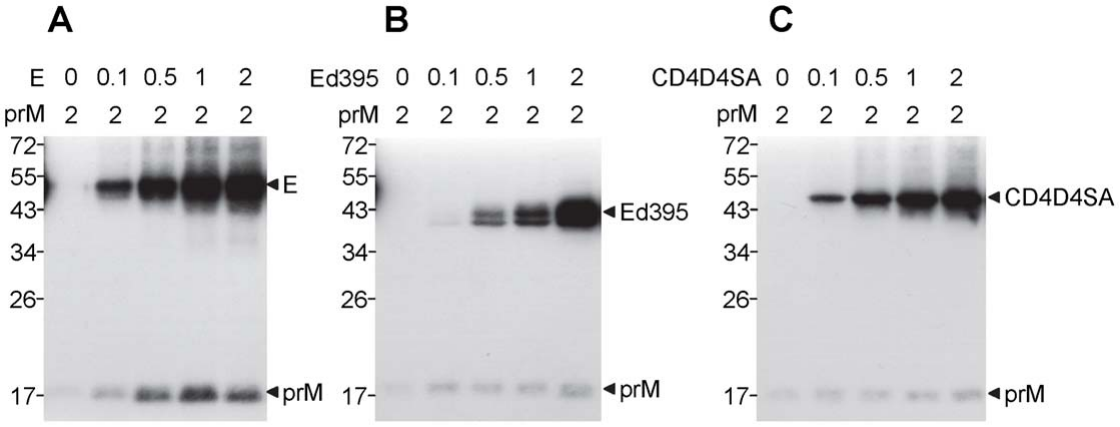
Effect of WT and mutant E proteins on the expression of prM protein. 293T cells were transfected with prM construct alone or prM construct and increasing amounts (0 to 2 µg) of WT E construct (A), E construct without the stem and anchor, Ed395 (B) or CD4D4SA construct containing the stem and anchor of E fused to the ectodomain of CD4 (C) [31]. Cell lysates collected at 48 h post-transfection were subjected to Western blot analysis using human serum of a confirmed dengue case [31], [32]. One representative experiment of three is shown. The size of molecular weight markers is shown in kDa. Arrow heads indicate prM, E, Ed395 or CD4D4SA protein.

Previous study of the C-terminal truncation of TBEV E protein revealed that ET1 and EH2 were involved in the heterodimerization of prM/E proteins [17], and raised the possibility that the MT1 and MH domains of prM protein might be involved in such interaction and affect the expression of prM protein. To investigate the effect of MT1, MH and E protein on the expression of prM protein, we expressed WT and a series of C-terminal truncation mutants of prM protein (Figure 1A) in the presence or absence of E protein. In the absence of E protein, prM protein alone expressed poorly (Figure 1B); all C-terminally truncated prM mutants did not expressed well. In contrast, co-transfection with E construct increased the expression of WT prM protein greatly and prMd147 protein slightly, suggesting that the effect of E protein on the expression of prM protein requires MT1 domain and MT2 domain is also involved. To further examine the effect of E protein on prM expression, increasing amounts of E construct were co-tranfected with prM construct. As shown in Figure 2A, a trend of increased expression of prM protein was observed as the amounts of E construct increased from 0.1 µg to 2 µg.

To investigate the domain of E protein required for the increased expression of prM protein, prM construct was co-transfected with WT E construct or each of the C-terminal truncation E constructs. As shown in Figure 1C, the expression of prM protein was good in the presence of WT E protein, decreased in the presence of Ed470 or Ed450 protein, and undetectable in the presence of Ed421 or Ed395 protein, suggesting that EH2 domain is critical for expression of prM protein and both ET2 and ET1 are also involved. As a comparison, WT E protein and C-terminally truncated E proteins expressed well without prM protein. The importance of EH2 domain was further confirmed by expressing prM and E proteins from the same construct including the WT prME and its C-terminally truncated constructs; the expression of prM protein was greatly reduced in the prMEd421 and prMEd395 constructs (Figures 1A and 1D). However, when EH2 domain was provided by a chimeric construct, CD4D4SA, which contained the ectodomain of CD4 and the stem-anchor regions (containing EH2 domain) of DENV4 E protein [31], it increased the expression of prM protein only slightly (Figure 2C). This finding suggested the importance of EH2 domain in the context of E protein for the increased expression of prM protein. Consistent with the importance of EH2, ectodomain of E protein alone (Ed395) increased the expression of prM protein only slightly (Figure 2B).

### EH2 domain is involved in maintaining the stability of prM protein

**Figure 3:**
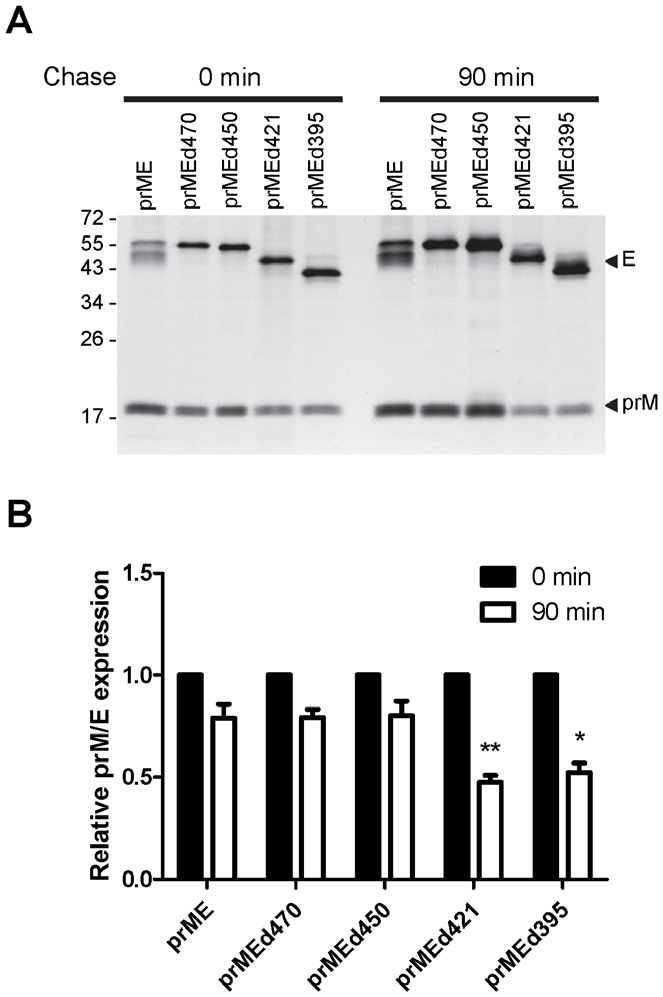
Effect of WT and C-terminally truncated E proteins on the expression and stability of prM protein by pulse-chase experiment. (A) 293T cells transfected with WT prME or prME constructs with C-teriminal truncation were pulsed for 20 min with [^35^S] methionine at 20 h post-transfection, and chased at 0 min and 90 min by immunoprecipitation with mixed human sera of confirmed dengue cases [33], followed by 12% PAGE as described in the Methods. One representative experiment of three is shown. The size of molecular weight markers is shown in kDa. Arrow heads indicate E and prM proteins. (B) Relative prM/E at 90 min was determined by the ratio of the intensity of prM band to truncated E band at 90 min divided by such ratio at 0 min as described in the Methods. *P = 0.002, **P = 0.009, two-tailed Mann-Whitney test.

To investigate if E protein affects the stability of prM protein, pulse-chase experiment was carried out for WT prME construct and a series of C-terminally truncated constructs. As shown in Figure 3, the expression of prM protein relative to E protein for mutants prMEd470 and prMEd450 during the chase at 90 min were comparable to that of WT. In contrast, the expression of prM protein relative to E protein for mutants prMEd421 and prMEd395 was reduced at 90 min (*P* = 0.002 and 0.009, respectively, two-tailed Mann-Whitney test), suggesting that in the absence of EH2 domain prM protein was not stable. Thus, EH2 is important for maintaining the stability of prM protein.

### ET1 and EH2 domains are involved in prM-E interaction

**Figure 4:**
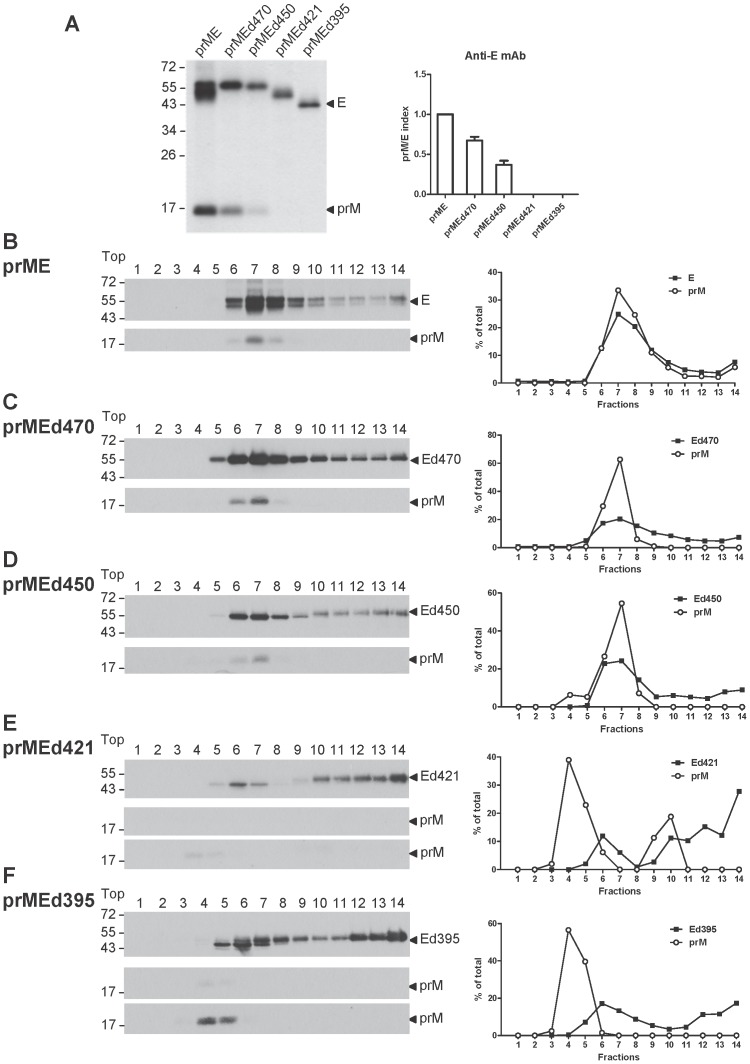
Interaction between prM protein and WT/C-terminally truncated E proteins by radioimmunoprecipitation assay and sucrose gradient sedimentation analysis. (A) 293T cells transfected with WT prME or prME constructs with C-terminal truncation were labeled with [^35^S] methionine at 20 h post-transfection, immunoprecipitated with an anti-E mAb FL0232, and subjected to 12% PAGE (left) as described in the Methods. One representative experiment of two is shown. The size of molecular weight markers is shown in kDa. Arrow heads indicate E and prM proteins. The prM/E index (right) was determined by the ratio of the intensity of prM band to truncated E band divided by such ratio of prM band to WT E band as described in the Methods. (B to F) Cell lysates derived from 293T cells transfected with WT prME or prME constructs with C-terminal truncation were subjected to 5 to 20% (wt/wt) sucrose gradient ultracentrifugation, and each of the 14 fractions was collected and subjected to Western blot analysis using serum from a confirmed dengue case [31], [32]. Long exposure of prM bands was shown for (E) and (F). The intensities of the E and PrM bands in each fraction were determined and presented as the percentage of total intensities of E and prM bands, respectively. One representative experiment of two is shown. The size of molecular weight markers is shown in kDa. Arrow heads indicate E and prM proteins.

To investigate the domains at the C-terminus of E protein that are involved in prM-E interaction and thus contribute to maintaining the stability of prM protein, radioimmunoprecipitation experiment using an anti-E mAb was carried out for WT prME and a series of C-terminal truncation mutants. As shown in Figure 4A, the prM/E index, which was the ratio of prM protein to mutant E protein relative to that of WT, was reduced for mutant prMEd450 and greatly reduced for mutants prMEd421 and prMEd395, suggesting that both ET1 and EH2 domains were involved in prM-E interaction.

We also performed a sucrose gradient sedimentation analysis using cell lysates derived from transfection of WT or each of the C-terminal truncation mutants. As shown in Figures 4B to 4C, the majority of E protein of WT and mutant prMEd470 was found in fractions 6 to 9 with the peak in fraction 7, which co-sedimented with that of prM protein except mutant prMEd470 had some E protein in fraction 5 not co-sedimented with prM protein, suggesting that prM-E heterodimerization was slightly affected by mutant prMEd470. In contrast, the peak of prM protein (fraction 4) of mutants prMEd421 and prMEd395 did not co-sediment with that of E protein, which had one peak in fraction 6 and another peak in fraction 14, suggesting that prM-E interaction was greatly affected (Figures 4E and 4F). For mutant prMEd450, the majority of prM protein was found in fractions 6 and 7, which co-sedimented with that of E protein, however, some E protein in fraction 5 did not co-sedimented with E protein, suggesting that prM-E interaction was affected (Figure 4D). This finding was generally consistent with that of radioimmunoprecipitation (Figure 4A). These results, taken together with that of the pulse-chase experiment, suggested that both ET1 and EH2 domains were involved in prM-E interaction and EH2 domain was also involved in maintaining the stability of prM protein.

Notably, the broad distribution of E protein in fractions 5 to 14 was in agreement with that of E1/E2 proteins of Hepatitis C virus, another flavivirus, under similar sucrose density analysis [36] and suggested the presence of E-E dimers or higher order complexes in high density fractions. We have carried out the sucrose gradient sedimentation analysis including Ed421, Ed395 and protein markers with known molecular weight, and found that the E protein in dense fractions (fractions 11 to 14) co-sedimented with a protein marker of 260 kDa (Figure S1, A to F), suggesting the formation of oligomeric form (such as tetramers) of E protein in cells. To rule out the formation of E aggregates in fraction 14 (of mutants prMEd421, prMEd395, Ed421 and Ed395), we have also recovered the E protein from fractions 14 and 6 (as a comparison), and conducted enzyme digestion with endo H and PNGaseF. An endo H-resistant pattern was found for truncated E protein in fraction 14 (prMEd421, prMEd395, Ed421 and Ed395) (Figure S1, H to K), suggesting that the truncated E protein in fraction 14 has passed the quality control machinery in ER and transported beyond trans-Golgi and therefore was unlikely to be aggregates retained in the ER or ER-associated degradation pathway [37].

### PrM protein and C-terminal truncation of E protein affect the recognition of E protein by mouse anti-E mAbs

**Figure 5:**
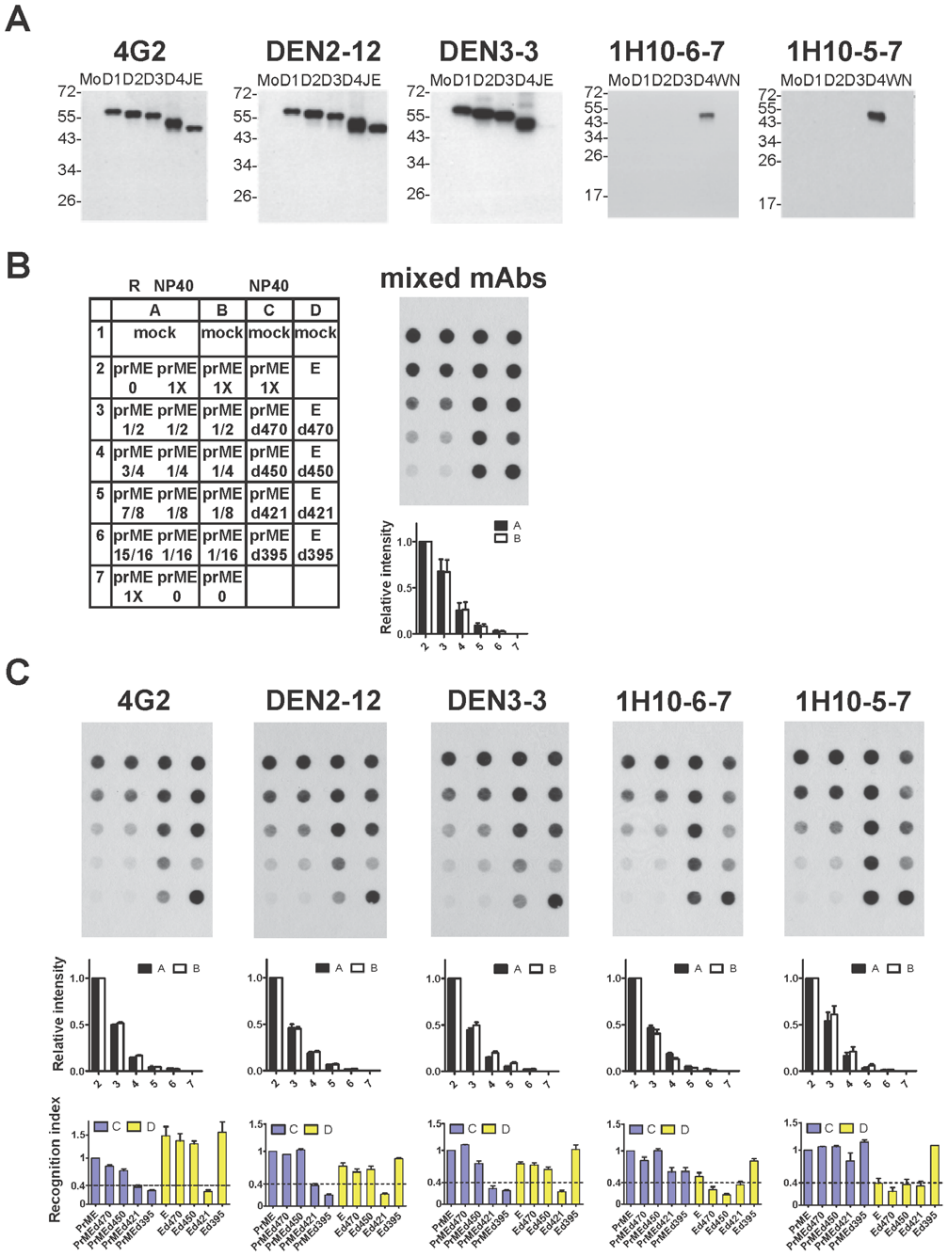
Effect of C-terminal E domains and prM protein on the recognition of E protein by different mouse anti-E mAbs. (A) Binding specificity of five mouse anti-E mAbs including GR (4G2 and DEN2-12), CR (DEN3-3), and DENV4 TS (1H10-6-7 and 1H10-5-7) mAbs. Western blot analysis was performed by using cell lysates derived from C6/36 cells infected with each of the four DENV serotypes, WNV or JEV. The size of molecular weight markers is shown in kDa. (B,C) Dot blot binding assay using these five mAbs to recognize WT E protein (expressed by prME), E protein alone and mutant E proteins containing C-terminal truncations (expressed by prME- or E-based constructs) in 1% NP40 lysis buffer (NP40). Layout of the dot blot assay and the binding by mixed mAbs are shown in (B). Decreasing amount of native WT E protein in 1% NP40 lysis buffer (column B) as well as mixtures containing decreasing amount of native WT E protein in 1% NP40 lysis buffer and increasing amount of denatured WT E protein in reducing (R) buffer (column A) were also included to control for exposure and sensitivity of the assay signal. Relative intensities of each dot in columns A (black bars) and B (white bars) were shown below each membrane. Recognition indices of each mAb to mutant E protein = [intensity of mutant E dot/intensity of WT E dot] (recognized by a mAb) divided by [intensity of mutant E dot/intensity of WT E dot] (recognized by mixed mAbs) were shown in blue bars (column C, in the presence of prM protein) and yellow bars (column D, in the absence of prM protein) below each membrane [33]. Data are mean and standard errors from two experiments.

Several C-terminal truncated E proteins in the presence or absence of prM protein have been included in the design of subunit vaccines [38]–[43]. To investigate if the conformation of E protein was affected by prM protein or by C-terminal truncation of E protein, we examined the E-binding activity for a panel of 5 mouse anti-E mAbs. Notably, the C-terminal domains of E protein (EH1, EH2, ET1 and ET2) do not contain the epitopes recognized by the anti-E mAbs in this study. As shown in Figure 5A, these mAbs recognized E protein only without significant background and were thus employed in a dot blot binding assay, in which cell lysates derived from transfection of different prME or E constructs were prepared in 1% NP40 lysis buffer, a mild non-ionic detergent to solubilize membrane protein. To exclude the possibility of overexposure, serially two-fold dilutions of cell lysates derived from WT prME construct were dotted on each membrane; a linear decrease in the intensity was found, suggesting that the assay signal is sensitive to serially two-fold decrease in the amount of E protein (Figures 5B and 5C, column B and white bars below each membrane). Moreover, only the E protein prepared in 1% NP40 lysis buffer (native E protein) but not that prepared in reducing buffer containing SDS and β-mercaptoethanol (denatured E protein) can be recognized by these mAbs (Figures 5B and 5C, rows 2 and 7 in column A of each membrane). A linear decrease in the intensity of dots was observed as the amount of native E protein decreased and that of denatured E protein increased, suggesting that the assay signal is sensitive to increasing proportion of denatured E protein (Figures 5B and 5C, column A and black bars below each membrane).

Compared with the intensity of the dot containing WT prME protein, the intensities of the dots containing E protein alone, C-terminal truncated E proteins, or C-terminal truncated prME proteins recognized by mixed mAbs were comparable (Figure 5B), suggesting that similar amounts of E protein were loaded in each dot. For the GR mAbs 4G2 and DEN2-12, the epitope of which involved fusion loop residues of domain II [33], the binding to mutants prMEd421, prMEd395 and Ed421 was greatly reduced (R.I.<0.4) when compared with the binding to WT prME protein, suggesting that EH2 is important for maintaining the conformation of E protein required for binding by 4G2 and DEN2-12 or preventing mis-folding of truncated E protein. Similar binding pattern was also observed for a CR mAb, DEN3-3 (Figure 5C).

For the TS mAb 1H10-5-7, the binding to E protein or most mutant E proteins (Ed470, Ed450 and Ed421, except Ed395) in the absence of prM protein was greatly reduced when compared to those in the presence of prM protein, suggesting that prM protein affects the conformation of E protein required for binding by 1H10-5-7 or prevents mis-folding of E protein (Figure 5C). Similar binding pattern was observed in another TS mAbs (1H10-6-7) except that its binding to E protein alone was not affected. Interestingly, Ed395 but not prMEd395 can be recognized well by these five mAbs, suggesting that in the absence of prM protein the ectodomain of E protein alone (Ed395) can fold well and preserve the epitopes recognized by these mAbs.

### PrM protein and C-terminal truncation of E protein affect the recognition of E protein by human anti-E mAbs

To further investigate if prM protein or C-terminal truncation of E protein affects the recognition of E protein by human anti-E mAbs, we examined the E-binding activity of 8 human anti-E mAbs (Figure S2A). For the six GR mAbs, the binding to mutants prMEd421 and Ed421 was greatly reduced (R.I.<0.4) in five when compared with that to WT prME protein, suggesting that EH2 is important for maintaining the conformation of E protein required for binding by these mAbs or preventing mis-folding of truncated E protein (Figure S2C). Another GR mAb DVD26.3 showed similar pattern with reduced binding to prMEd421 and Ed421, though the R.I. was slightly above the cutoff of 0.4.

For the TS mAbs DVD9.8 and DVD9.9, the binding to E protein or most of the C-terminal truncated E proteins (Ed470, Ed450 and Ed421, except Ed395) in the absence of prM protein was greatly reduced when compared with that to WT prME protein (Figure S2C), suggesting that prM protein affects the conformation of E protein required for binding by these two TS mAbs. In the presence of prM protein, the binding to mutants prMEd421 and prMEd395 was also reduced (R.I.<0.4) when compared with that to WT prME protein, suggesting that EH2 and EH1 are important for maintaining the conformation of E protein required for binding by these two mAbs or preventing mis-folding of truncated E protein. Notably, Ed395 but not prMEd395 can be recognized well by these 8 human mAbs, suggesting that in the absence of prM protein Ed395 can fold well and preserve the epitopes recognized by these mAbs.

**Figure 6:**
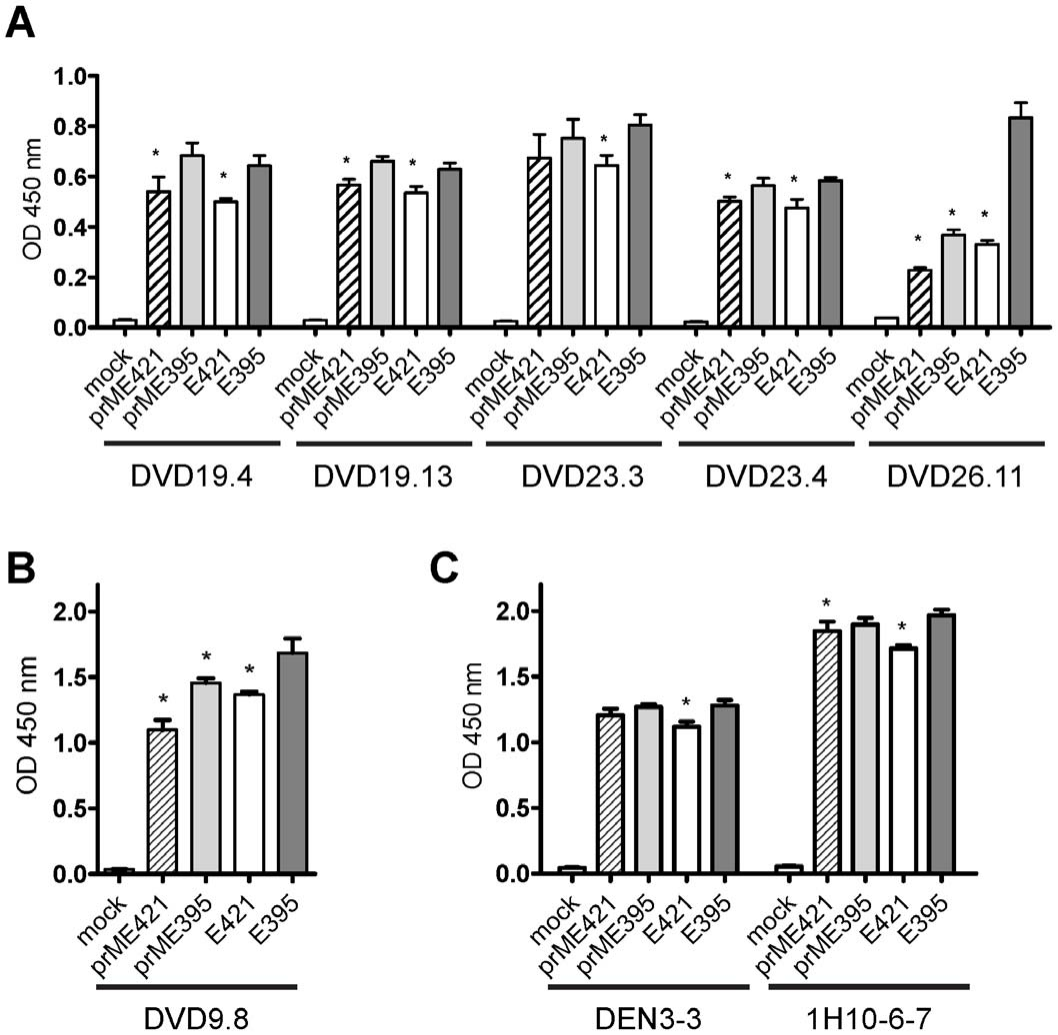
Binding of C-terminally truncated E proteins in extracellular fluid to different human and mouse anti-E mAbs by capture ELISA. (A) Human GR anti-E mAbs. (B) Human TS anti-E mAb. (C) Mouse CR anti-E mAb, DEN3-3, and TS anti-mAb, 1H10-6-7. Comparable amounts (0.6 ng each) of truncated E protein derived from prMEd421, prMEd395, Ed421 or Ed395 were added to 96 well plate pre-coated with mixed mouse mAbs (for testing human mAbs) or human dengue-immune serum (for testing mouse mAbs), followed by addition of each human or mouse mAb and secondary antibody as described in Methods. Data are means and standard errors of quadricates from one representative experiment of two. *P = 0.03, two-tailed Mann-Whitney test.

We also carried out a capture-ELISA to study the recognition of C-terminally truncated E proteins (prMEd421, prMEd395, Ed421, Ed395), most of which affected the binding in the dot blot binding assay, in extracellular fluid by human and mouse mAbs. For most of the human GR mAbs tested, the binding to Ed395 was comparable to that to prMEd395 but better than to prMEd421 and Ed421 (Figure 6A); this is generally in agreement with the results of dot blot binding assay. For the human TS mAb (DVD9.8) tested, its binding to Ed395 was better than to prMEd395, prMEd421 and Ed421 (Figure 6B), which is in agreement with the results of dot blot binding assay. For the mouse GR (DEN3-3) and TS (1H10-6-7) mAbs tested, the binding to Ed395 was better than to prMEd421 and Ed421 (Figures 6C); this is also consistent with the results of dot blot binding assay. Together, the capture-ELISA revealed that Ed395 in the extracellular fluid but not other truncated E proteins (prMEd395, prMEd421 and Ed421) can be recognized well by all 6 human and 2 mouse mAbs tested.

### E protein affects the recognition of prM protein by human anti-prM mAb

**Figure 7:**
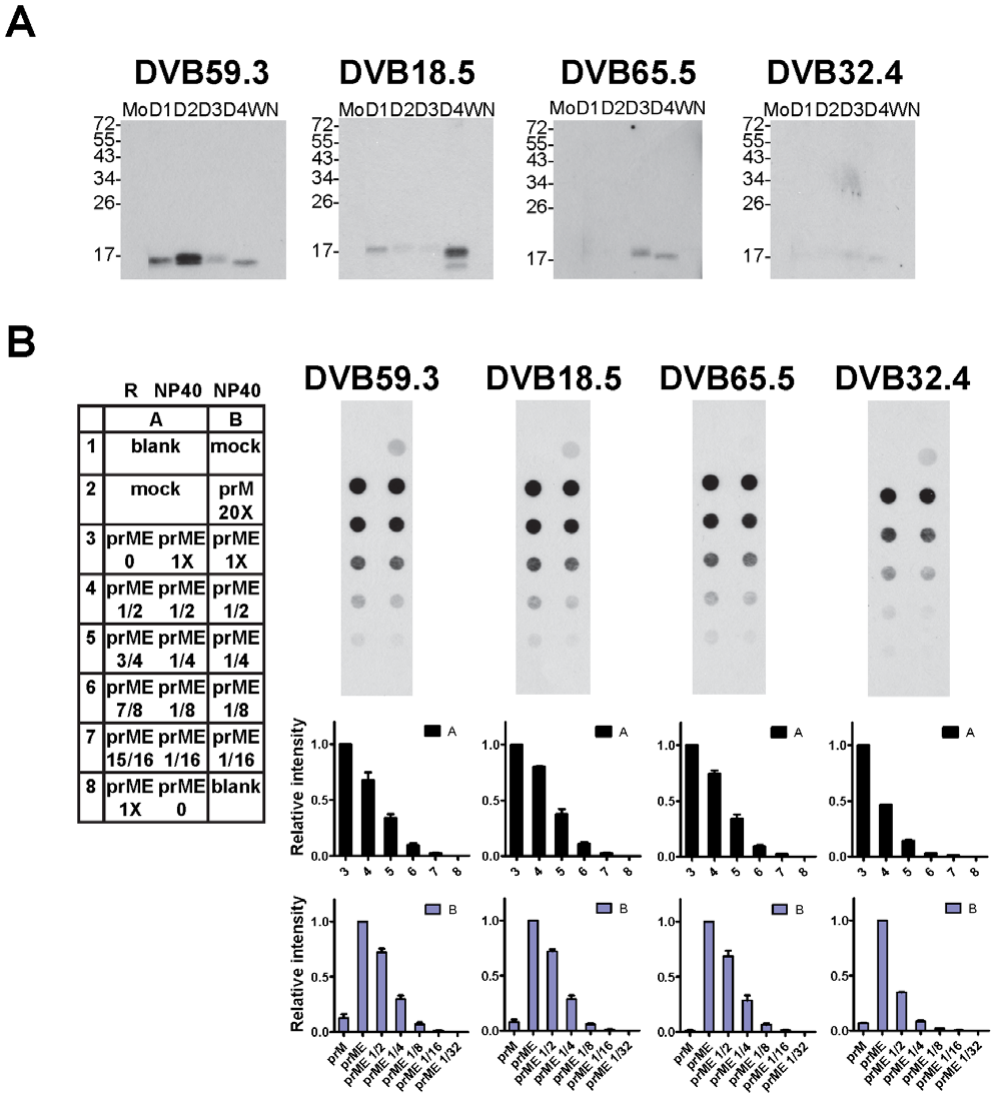
Effect of E protein on the recognition of prM protein by human anti-prM mAbs. (A) Binding specificity of 4 human anti-prM mAbs including 2 CR (DVB59.3 and DVB18.5) and 2 sCR (DVB65.5 and DVB32.4) mAbs was determined as in Figure 5. (B) Dot blot binding assay using these 4 mAbs to recognize prM protein in the presence (expressed by prME) or absence (expressed by prM) of E protein in 1% NP40 lysis buffer (NP40). Decreasing amount of prM protein (expressed by prME) in 1% NP40 lysis buffer (column B, rows 3 to 7) and mixtures containing decreasing amount of prM protein in 1% NP40 lysis buffer and increasing amount of denatured prM protein in reducing (R) buffer (column A) were included to control for exposure and sensitivity of the assay signal, respectively. Twenty times more cell lysates derived from transfection of prM alone were loaded. Relative intensities of each dot in column A (black bars) and column B (blue bars) were shown below each membrane. Data are mean and standard errors from two experiments.

To investigate the effect of E protein on the conformation of prM protein, we carried out a similar dot blot binding assay by using cell lysates derived from transfection of prME or prM construct alone to examine the prM-binding activity of a panel of 4 human anti-prM mAbs (Figure 7A) [35]. It should be noted that C-terminal truncation of E protein (especially EH2) greatly affect the expression and stability of prM protein (Figures 1D and 3), therefore the effect of C-terminal E domains on the recognition of prM protein by anti-prM mAbs were not tested. Controls to rule out the possibility of overexposure and to demonstrate that the assay signal is sensitive to increasing proportions of denatured prM protein were carried out (Figure 7B, rows 3 to 8 in column A of each membrane) as described in Figure 5. Since E protein affected the stability of prM protein, 20-fold more cell lysates derived from transfection of prM construct alone were loaded compared with those derived from WT prME. As shown in Figure 7B, in the absence of E protein the intensity of prM dots recognized by three mAbs (DVB59.3, DVB18.5 and DVB32.4) was comparable to that of 1∶8 or 1∶4 dilution of prM protein in the presence of E protein (rows 2, 5 and 6 in column B), confirming the instability of prM protein in the absence of E protein. Notably, anti-prM mAb DVB65.5 cannot recognize prM dot in the absence of E protein, suggesting that E protein affects the conformation of prM protein required for binding by this mAb.

## Discussion

In this study, we investigated the roles of DENV E protein on the expression, stability and conformation of prM protein and how prM protein and C-terminal truncation of E protein affect the conformation of E protein. In the absence of E protein, prM protein did not express well. In the presence of E protein, the expression of prM protein increased in a dose-dependent manner. Pulse-chase experiment suggested EH2 is important for maintaining the stability of prM protein. Moreover, E protein affected the recognition of prM protein by anti-prM mAbs. To our knowledge, this is the first study reporting that DENV prM protein alone expresses poorly and a domain of E protein (EH2) can affect the stability and expression of prM protein, a chaperone of E protein. This is in contrast to what has been reported for TBEV prM protein, which was stable and expressed well by itself [25]. Dot blot binding assay revealed that prM protein and C-terminal truncation of E protein affect the recognition of E protein by several mouse and human anti-E mAbs. These findings not only add to our understanding of the interaction between DENV prM and E proteins but also have implication for future design of subunit dengue vaccines.

A recent crystallographic study of a recombinant DENV2 prM/E protein complex revealed the heterodimeric interaction between the N-terminus of prM protein (residues 1 to 81 of pr peptide) and E protein ectodomain at high resolution [44]. Our radioimmunoprecipitation and sucrose gradient sedimentation analysis of a series of C-terminally truncated prME constructs of DENV4 revealed that ET1 and EH2 rather than the ectodomain of E protein were critical for prM-E interaction (Figure 4). Similar finding has been reported for TBEV by radioimmunoprecipitation analysis of C-terminally truncated prME constructs [17]. These observations suggest that ET1 and EH2 probably contribute to prM-E interaction greater than the ectodomain of E protein. Cryo-EM study of DENV2 virions at high resolution revealed that the corresponding double membrane anchors of E protein (ET1, ET2) and M protein (MT1, MT2) were packed together and ET1 was next to MT1 [18], it is conceivable that the interaction between ET1 and MT1 within the membrane contributed significantly to the prM-E interaction. In addition, the EH2 and MH are partially buried in the outer leaflet of membrane [18]. It is possible that EH2 could interact directly with MH or other region of prM protein (such as residues 82 to 112) not depicted clearly by the crystal structure of recombinant prM/E protein complex [44].

In this study, we employed a dot blot binding assay using cell lysates (of different prME or E transfectants) prepared in 1% NP40 lysis buffer without SDS or boiling, a condition similar to RIPA lysis buffer containing non-ionic detergent, to preserve the conformation of membrane protein. In addition to the controls to exclude the possibility of overexposure (Figures 5B and 5C, column B of each membrane), we also prepared mixtures containing different amounts of native E protein (in 1% NP40 lysis buffer) and denatured E protein (in reducing buffer) to assess the recognition by different anti-E mAbs (Figures 5B and 5C, column A of each membrane). It is worth noting that the conformation of E protein examined in our dot blot binding assay was that of E protein in heterodimer with prM protein and might not be the same as that in the context of particles. Since most of the C-terminal truncation constructs (except prMEd470) did not form VLPs well (data not shown), a finding consistent with a previous report in TBEV [17], the binding of these C-terminal truncated E proteins in the context of particles was not examined.

**Figure 8:**
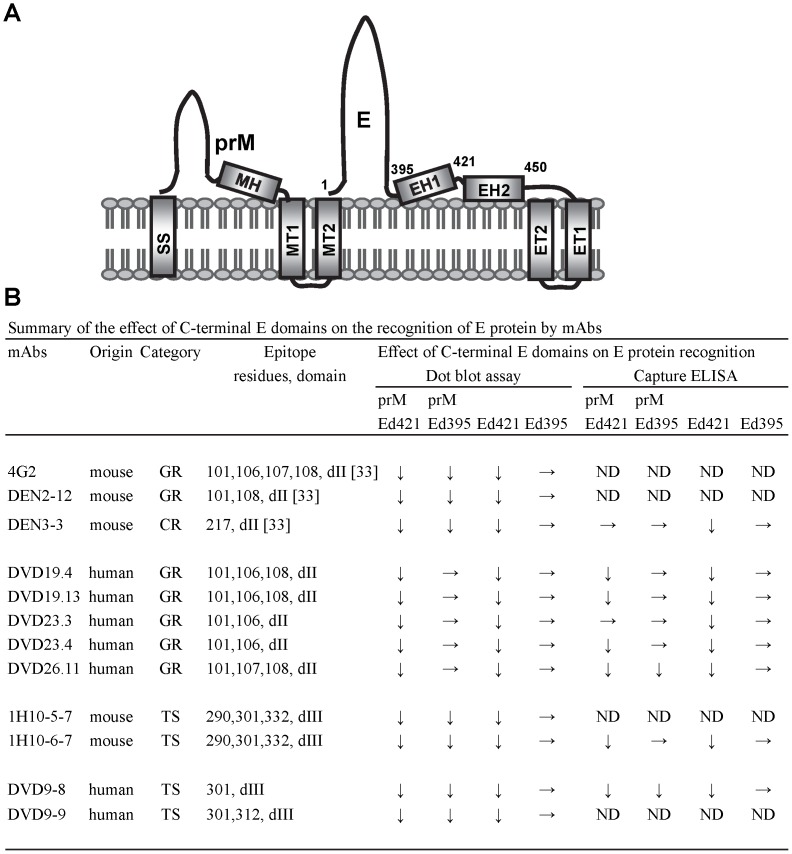
Schematic drawing of prM/E proteins after synthesis and summary of the effect of C-terminal E domains on the recognition of E protein by mAbs. (A) Schematic drawing of prM/E proteins on ER membrane after synthesis. The topology of the stem (MH, EH1, EH2) and anchor (MT1, MT2, ET1, ET2) regions on membrane were based on a cryo-EM study of DENV virions at high resolution [18]. The ectodomains of prM and E proteins were drawn disproportionately. SS: signal sequence. The numbers of E residues between domains were shown. (B) Summary of the effect of C-terminal E domains on the recognition of E protein by mAbs based on dot blot assay and capture ELISA. Epitope residues were determined by binding assays involving a panel of 67 alanine mutants of predicted surface-exposed E residues as described previously [33]. ↓ indicates reduced binding (R.I.<0.4 in dot blot assay or *P*<0.05 in capture ELISA) to mutant E proteins (prMEd421, prMEd395, Ed421); →indicates binding was not reduced. ND, not done.

Figure 8 summarizes the effect of prM protein and C-terminal E truncations on the recognition of E protein by mAbs based on dot blot binding assay and capture-ELISA. Truncation of EH2 or EH1 greatly affected the binding of E protein by two mouse GR mAbs (4G2 and DEN2-12), which recognized fusion loop residues of domain II [33], whereas truncation of EH2 but not EH1 greatly affected E protein binding by five human GR mAbs (DVD19.4, DVD19-13, DVD23.3, DVD23.4 and DVD26.11), which recognized similar fusion loop residues (Figure 8B). The reduced binding to prMEd421 and Ed421 suggests that EH2, buried in the outer leaflet of membrane, is important for proper folding or maintaining the conformation of E protein (prMEd421, Ed421) required for recognition by these mAbs. Notably, our previous study found that proline substitutions introduced to EH1 and EH2 did not affect the recognition by several mouse anti-E mAbs, suggesting differential effects of substitution and truncation [45]. The reduced binding to prMEd395 by mouse GR mAbs but not by human GR mAbs suggests that mouse mAbs is sensitive to different conformations of Ed395 in the presence or absence of prM protein. Alternatively, prM protein may interfere with the binding of mouse anti-E mAbs to prMEd395 through steric hindrance. In the absence of prM protein the binding of E protein (except Ed395) by four anti-E TS mAbs was reduced, suggesting that prM protein is important for maintaining the conformation of E protein (except Ed395) required for recognition by these anti-E mAbs.

Interestingly, the ectodomain of E protein alone (Ed395) could be recognized well by all the anti-E mAbs tested, suggesting that the ectodomain of E protein alone can fold well by itself and preserve the conformation and epitopes recognized by different anti-E mAbs. Whether Fd395 can also be recognized by other newly discovered anti-E mAbs remains to be tested; our findings suggest that Ed395 among different C-terminal truncated E proteins is a potential subunit immunogen mimicking the native conformation of E protein. Consistent with this interpretation, a previous study comparing the immunogenicity of truncated and full-length E protein with or without prM protein (D1E80, D1ME80, D1ME92 and D1ME100) by DNA vaccines in mice revealed that only D1ME100 (corresponding to our WT prME) and D1E80 (corresponding to our Ed395) induced neutralizing antibodies [38]. Another study used a series of C-terminally truncated DENV4 E proteins derived from recombinant vaccinia virus to immunize mice and reported that 79%-RKG construct (corresponding to truncation at residue 394) was most immunogenic, whereas 81% and 100% constructs (corresponding to truncation at residues 399 and 436, respectively) induced very low or no antibodies [39]. Similarly, recombinant WNV E protein with truncation at residue 406 (corresponding to our DENV Ed395 construct) can induce neutralizing antibodies and showed protection in mice and hamsters [40]. Other studies expressing recombinant DENV E protein with truncation at residue 421, 424, 442 or 437 (in DNA vaccine) showed only partial protection in mice or monkeys [41]–[43]. It should be noted that a previous study reported that inactivated whole TBEV virus and DNA vaccine encoding prM/E proteins induced higher neutralizing antibodies and better protection in mice than DNA vaccine encoding truncated E or truncated prM/E proteins (corresponding to our DENV Ed395 or prMEd395) [46]. A recent study of DENV suggested the importantce of mAbs that recognize quaternary structure [47]. Therefore, further studies to compare the potency of Ed395 and inactivated whole DENV as vaccines are warranted.

While several tetravalent live-attenuated candidate DENV vaccines have moved to Phase II or III clinical trials, a major challenge is the difficulties in achieving balanced neutralizing antibodies against all four serotypes due to dominant viremia by one or two serotypes resulting from inter-serotype interference and the risk of ADE mediated by cross-reactive non-neutralizing antibodies [13], [14], [48]. Several subunit vaccines including different recombinant proteins and DNA vaccines are under development to avoid viral interference and/or better present the neutralizing epitopes [13], [14]. Previously, cross-reactive non-neutralizing or poorly neutralizing anti-E antibodies were thought to be the major player of ADE; recent studies revealed that anti-prM mAbs did not neutralize DENV well and potently promote infectivity by ADE [27]–[29]. These studies suggest that anti-prM responses should be minimized in future dengue vaccines. Since prM protein was reported as a chaperone for proper folding of E protein [24], [25], how to design subunit vaccines presenting E protein in its native conformation in the absence of prM protein is critical. Because secreted E protein is preferred for subunit vaccine preparation, various C-terminal truncations of E protein to remove transmembrane anchor have been designed [38]–[43]. Based on the analysis of binding of mAbs to a series of C-terminally truncated E proteins in the presence or absence of prM protein, our findings that the ectodomain of E protein alone (Ed395) can be recognized well by all the anti-E mAbs tested suggest it could be a potential subunit immunogen that preserves the conformation of E protein without inducing anti-prM response.

## Supporting Information

Figure S1 **Sucrose gradient sedimentation analysis of WT and C-terminally truncated E proteins and glycosylation patterns of E proteins in fractions 14 and 6.** (A to F) Cell lysates derived from 293T cells transfected with WT prME or truncated constructs (prMEd421, prMEd395, Ed421 and Ed395) as well as protein markers were subjected to 5 to 20% (wt/wt) sucrose gradient ultracentrifugation, and each of the 14 fractions was collected and subjected to Western blot analysis using a dengue-immune serum [31], [32]. E proteins in dense fractions (fractions 11 to 14) co-sedimented with a protein marker of 260 kDa. (G to K) Aliquots of total cell lysates and fractions 14 and 6 derived from transfection of WT prME or each of the truncated constructs were digested with endo H or PNGase F and subjected to Western blot analysis using a dengue-immune serum. The size of molecular weight markers is shown in kDa. Arrow heads indicate WT or truncated E proteins and their deglycosylated (dg) forms.
(TIF)

Figure S2 **Effect of C-terminal E domains and prM protein on the recognition of E protein by different human anti-E mAbs.** (A) Binding specificity of 8 human anti-E mAbs including GR (DVD19.4, DVD19.13, DVD23.3, DVD23.4, DVD26.3 and DVD26.11) and DENV4 TS (DVD9.8 and DVD9.9) mAbs was determined as in Figure 5. (B,C) Dot blot binding assay using these 8 mAbs to recognize WT E protein (expressed by prME), E protein alone and mutant E proteins containing C-terminal truncations (expressed by prME- or E-based constructs) in 1% NP40 lysis buffer (NP40). The controls and data presentation were as in Figure 5.
(TIF)

Table S1 **Sequences of the primers for PCR and cloning in this study.**
(DOC)
