# Supplementary figures and images for: C-Terminal Helical Domains of Dengue Virus Type 4 E Protein Affect the Expression/Stability of prM Protein and Conformation of prM and E Proteins
Source: PLoS One. 2012 Dec 26;7(12):e52600. doi: 10.1371/journal.pone.0052600 (PMC3530441; doi:10.1371/journal.pone.0052600)

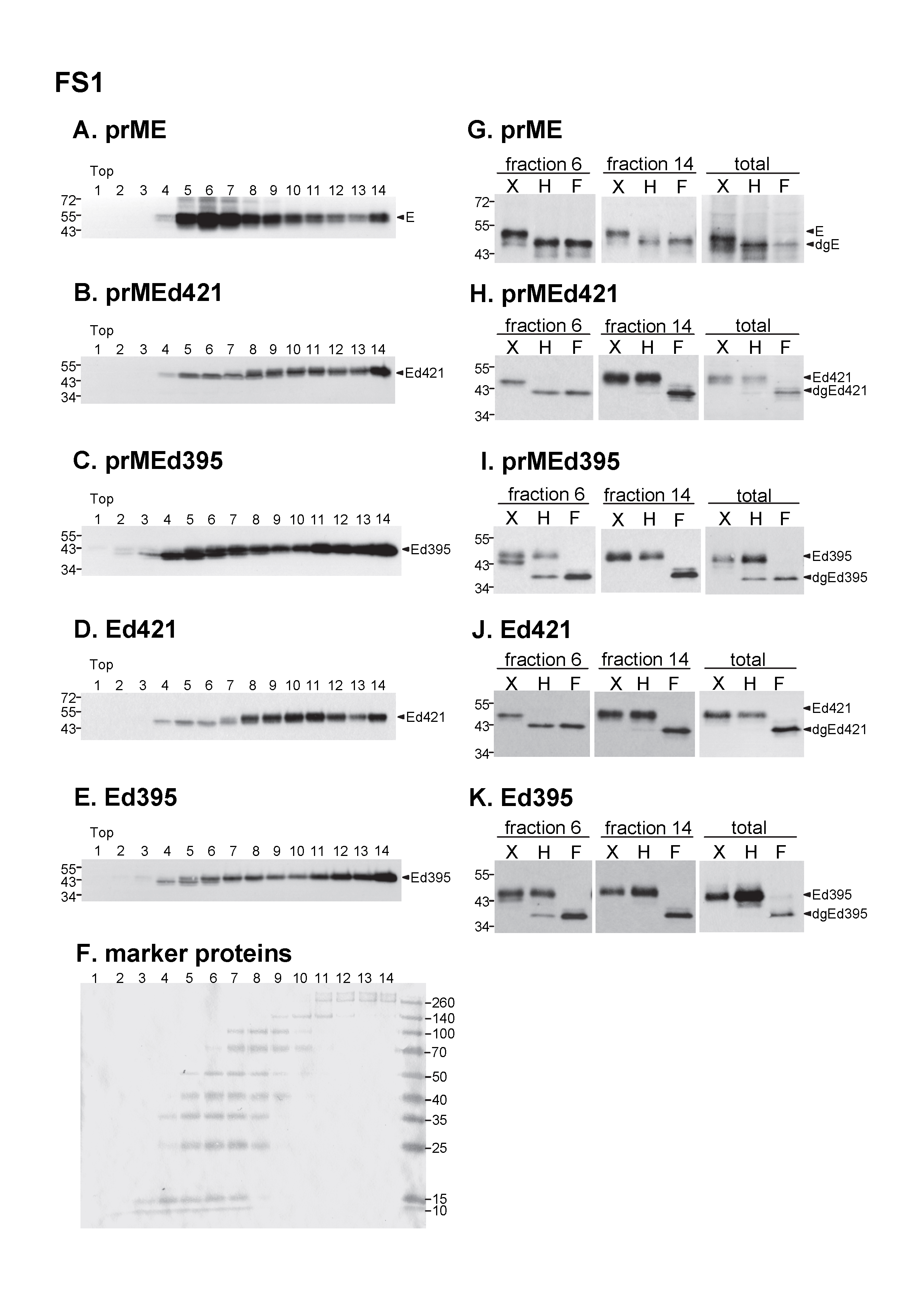

Supplement: Figure S1 — Sucrose gradient sedimentation analysis of WT and C-terminally truncated E proteins and glycosylation patterns of E proteins in fractions 14 and 6. (A to F) Cell lysates derived from 293T cells transfected with WT prME or truncated constructs (prMEd421, prMEd395, Ed421 and Ed395) as well as protein markers were subjected to 5 to 20% (wt/wt) sucrose gradient ultracentrifugation, and each of the 14 fractions was collected and subjected to Western blot analysis using a dengue-immune serum [31], [32]. E proteins in dense fractions (fractions 11 to 14) co-sedimented with a protein marker of 260 kDa. (G to K) Aliquots of total cell lysates and fractions 14 and 6 derived from transfection of WT prME or each of the truncated constructs were digested with endo H or PNGase F and subjected to Western blot analysis using a dengue-immune serum. The size of molecular weight markers is shown in kDa. Arrow heads indicate WT or truncated E proteins and their deglycosylated (dg) forms. (TIF) [file pone.0052600.s001.tif]

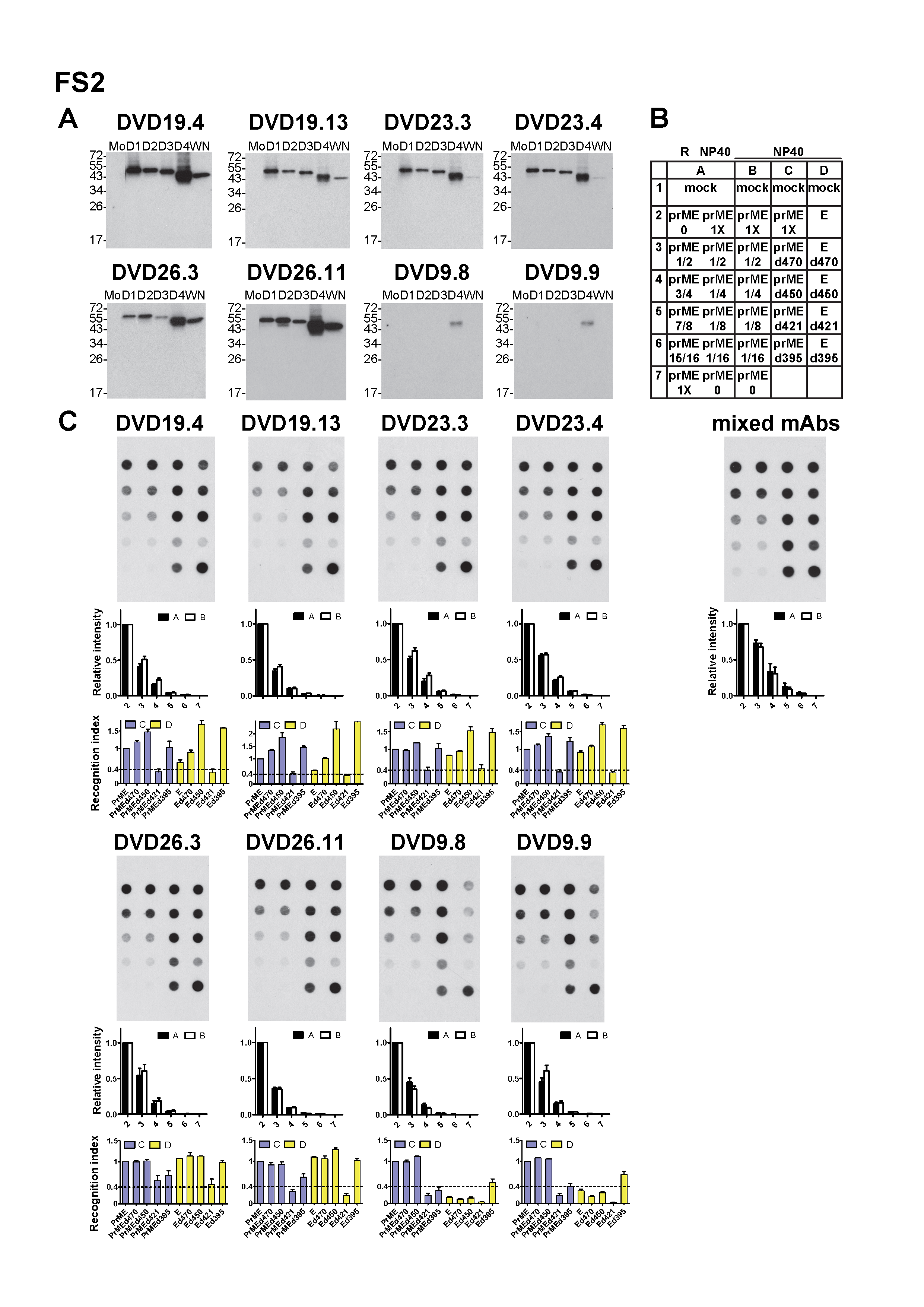

Supplement: Figure S2 — Effect of C-terminal E domains and prM protein on the recognition of E protein by different human anti-E mAbs. (A) Binding specificity of 8 human anti-E mAbs including GR (DVD19.4, DVD19.13, DVD23.3, DVD23.4, DVD26.3 and DVD26.11) and DENV4 TS (DVD9.8 and DVD9.9) mAbs was determined as in Figure 5. (B,C) Dot blot binding assay using these 8 mAbs to recognize WT E protein (expressed by prME), E protein alone and mutant E proteins containing C-terminal truncations (expressed by prME- or E-based constructs) in 1% NP40 lysis buffer (NP40). The controls and data presentation were as in Figure 5. (TIF) [file pone.0052600.s002.tif]
