# Supplementary material for: C-Terminal Helical Domains of Dengue Virus Type 4 E Protein Affect the Expression/Stability of prM Protein and Conformation of prM and E Proteins
Source: PLoS One. 2012 Dec 26;7(12):e52600. doi: 10.1371/journal.pone.0052600 (PMC3530441; doi:10.1371/journal.pone.0052600)
Supplement: Table S1 — Sequences of the primers for PCR and cloning in this study. (DOC) [file pone.0052600.s003.doc]

**Table S1.** Sequences of the primers for PCR and cloning in this study

| Primers | Sequences |
| --- | --- |
| d4prM91BNotI | 5'- CTTCGAGCGGCCGCTTAGCGCTTCTCTCGTCTCCG -3' |
| d4prM110BNotI | 5'- CTTCGAGCGGCCGCTTACCATGTCTCAGCTCTTGTTT -3' |
| d4prM129BNotI | 5'- CTTCGAGCGGCCGCTTATCTGAGTATCCAGCTCTCTA -3 |
| d4prM147BNotI | 5'- CTTCGAGCGGCCGCTTATGTTTGCCCAATCATATAAGC -3' |
| d4prM166BNotI | 5'- CTTCGAGCGGCCGCTTATCCGTAGGATGGGGCGAC -3' |
| d4KpnSS-A | 5'- CTTGGTACCGCCGCCGCCATGACGATAACATTGCTGTGCTTG -3' |
| d4KpnSSEA | 5'- CTTGGTACCGCCGCCGCCATGCAGCGAACTGTCTTCTTTGTC -3' |
| d4E395BNotI | 5'- CTTCGAGCGGCCGCTTACCCTTTCCTGAACCAATGG -3' |
| d4E421BNotI | 5'- CTTCGAGCGGCCGCTTAATCCCAAGCTGTTTCACCTA -3' |
| d4E450BNotI | 5'- CTTCGAGCGGCCGCTTATCCTCCAAACATGGTTGTATA -3' |
| d4E470BNotI | 5'- CTTCGAGCGGCCGCTTACGAGTTCGTGCCAATCCA -3' |
| d4E495BNotI | 5'- CTTCGAGCGGCCGCTCAACTAATTATGCTTGAACTGTGAAGCCC -3' |

To generate individual prM and E expression constructs, PCR was performed using the primers (d4KpnSS-A plus d4prM166BNotI, and d4KpnSSEA plus d4E495BNotI, respectively) and prME as template, and cloned back to pCB vector by KpnI and NotI sites. To generate the C-terminal truncation constructs of E alone, PCR was performed using the primers (d4KpnSSEA plus d4E495BNotI, d4E470BNotI, d4E450BNotI, d4E421BNotI, or d4E395BNotI) and prME as template. To generate the C-terminal truncation constructs of prME, the above truncated E constructs were cloned back to prME by BstEII and NotI sites. To generate the C-terminal truncation constructs of prM alone, PCR was performed using the primers (d4KpnSS-A plus d4prM166BNotI, d4prM147BNotI, d4prM129BNotI, d4prM110BNotI, or d4prM91BNotI) and prM construct as template.
